# Supplementary material for: Making the match and breaking it: values, perceptions, and obstacles of trainees applying into physician-scientist training programs
Source: BMC Med Educ. 2023 Oct 12;23:763. doi: 10.1186/s12909-023-04736-w (PMC10571326; doi:10.1186/s12909-023-04736-w)
Supplement: Supplementary file 1 — Additional file 1. IM Physician-Scientist Training Program (PSTP) Applicant Survey (2021 - 2022). [file 12909_2023_4736_MOESM1_ESM.pdf]

IM Physician-Scientist Training Program (PSTP) Applicant Survey (2021 - 2022)

In this survey, we hope to capture the applicant experience among physician-scientist trainees seeking to enter research-oriented Internal Medicine residency programs, or physician-scientist training programs (PSTPs). Thus, this survey should ONLY be completed by applicants who applied to AT LEAST 1 INTERNAL MEDICINE PHYSICIAN-SCIENTIST TRAINING PROGRAM (PSTP)** during the 2021-2022 cycle.

**Physician-scientist training programs (PSTPs) refer to residency tracks that "integrate clinical training in internal medicine RESIDENCY AND SUBSPECIALTY WITH RESEARCH ACTIVITIES over a six to seven-year period of training time." For other details, please see: https://students-residents.aamc.org/md-phd-dual-degree-training/training-physician-scientist-internal-medicine
----

Your participation in this research study is strictly voluntary. You may choose not to participate. If you decide to participate in this research survey, you may withdraw at any time. If you decide not to participate in this study or if you withdrawl from participating at any time, you will not be penalized.

The procedure involves filling an online survey that will take approximately 10 minutes or less to finish. Your responses will be confidential and we do not collect identifying information such as your name, email address or IP address. The survey questions will be about your application and interview experiences to PSTPs during the 2021-2022 residency interview cycle.

We will do our best to keep your information confidential. All data is stored in a password protected electronic format. To help protect your confidentiality, the surveys will not contain information that will personally identify you. The results of this study will be reported as aggregate values only, and for scholarly purposes only.

If you have any questions about the research study, please contact Mark Pepin (pepinme@gmail.com). This research involves anonymous survey responses, and is exempt from IRB procedures for research involving human subjects.

ELECTRONIC CONSENT: Please select your choice below.

Clicking on the "agree" button below indicates that:

• you have ready the above information
• you voluntarily agree to participate
• you are at least 18 years of age

If you do not wish to participate in the research study, please decline participation by clicking on the "I disagree" button.

1.

Mark only one oval.

- ( ) I agree
- ( ) I disagree

"Tell us a bit about yourself" (1/6)

2. Expected Degrees (before residency)

Mark only one oval.

- ( ) MD
- ( ) MD, PhD
- ( ) MD, MS
- ( ) MD, MPH
- ( ) MD + Other Graduate

3. In which state did you attend medical school?

Mark only one oval.

- ( ) I attended outside of the continental U.S.
- ( ) Alabama
- ( ) Alaska
- ( ) Arizona
- ( ) Arkansas
- ( ) California

- Colorado
- Connecticut
- Delaware
- Florida
- Georgia
- Hawaii
- Idaho
- Illinois
- Indiana
- Iowa
- Kansas
- Kentucky
- Louisiana
- Maine
- Maryland
- Massachusetts
- Michigan
- Minnesota
- Mississippi
- Missouri
- Montana
- Nebraska
- Nevada
- New Hampshire
- New Jersey
- New Mexico
- New York
- North Carolina
- North Dakota
- Ohio

- Oklahoma
- Oregon
- Pennsylvania
- Rhode Island
- South Carolina
- South Dakota
- Tennessee
- Texas
- Utah
- Vermont
- Virginia
- Washington
- West Virginia
- Wisconsin
- Wyoming

4. Were any of your degrees obtained overseas?

Mark only one oval.

- Yes
- No

5. To which gender do you identify?

Mark only one oval.

- Female
- Male
- Transgender Female
- Transgender Male
- Prefer not to share
- Other: ____________________

6. Are you of Hispanic, Latino, or Spanish origin?

Mark only one oval.

- Yes
- No
- Prefer not to share

7. How would you describe your race?

Mark only one oval.

- American Indian or Alaska Native
- Asian
- Black or African American
- Native Hawaiian or Other Pacific Islander
- White
- Prefer not to share
- Other: ____________________

8. Are you currently living with a disability?

Mark only one oval.

- Yes
- No
- Prefer not to share

9. Step 1 Score Range (will not be shared)

Mark only one oval.

- < 210
- 210 - 220
- 220 - 230
- 230 - 240
- 240 - 250
- 250 - 260
- > 260

10. Step 2 CK Score Range (will not be shared)

Mark only one oval.

- < 210
- 210 - 220
- 220 - 230
- 230 - 240
- 240 - 250
- 250 - 260
- > 260

11. Academic Honor Society membership

Check all that apply.

- [ ] Alpha Omega Alpha (AOA) member
- [ ] Gold Humanism Honor society member
- [ ] My medical school does not have AOA and/or Gold Humanism Society

12. How many first-authored, peer-reviewed manuscripts did you have when you applied?

____

13. How many peer-reviewed manuscripts did you have when you applied?

____

14. Did you obtain extramural (e.g. NIH F-series) funding during graduate non-medical (PhD, MPH etc...) training?

Mark only one oval.

- Yes
- No
- N/A

15. What MOST attracted you to apply for a PSTP?

Mark only one oval.

- Protected research time
- Guaranteed fellowship
- Guaranteed research funding
- Physician-scientist community
- Structured mentoring
- Opportunity to "Fast-track" into postdoctoral research
- Other: _______________

16. How important was it for PSTPs to offer a GUARANTEED FELLOWSHIP position?

Mark only one oval.

	1	2	3	4	5	
Not Important						Essential

17. ^ Why / why not?

18. How important is STRUCTURED CAREER MENTORING within PSTPs? (via PSTP-organized events, scheduling, etc...)

Mark only one oval.

	1	2	3	4	5	
Not Important	⬭	⬭	⬭	⬭	⬭	Essential

19. How important is it for PSTPs to offer FINANCIAL BENEFITS (research, stipend, etc...)?

Mark only one oval.

	1	2	3	4	5	
Not Important	⬭	⬭	⬭	⬭	⬭	Essential

20. How important is it to have a community of PHYSICIAN-SCIENTIST TRAINEES at the same institution?

Mark only one oval.

	1	2	3	4	5	
Not Important	⬭	⬭	⬭	⬭	⬭	Essential

21. What is your intended subspecialty?

Mark only one oval.

- ⬭ Acute Care Medicine / Pulmonology
- ⬭ Cardiology
- ⬭ Endocrinology
- ⬭ Geriatrics
- ⬭ Gastroenterology
- ⬭ Hematology/Oncology
- ⬭ Infectious Diseases
- ⬭ Nephrology
- ⬭ Rheumatology
- ⬭ Unsure
- ⬭ None
- ⬭ Other: ____________________

The Application - Your Perspective (2/6)

Physician-scientist training programs (PSTPs) refer to residency tracks that "integrate clinical training in internal medicine RESIDENCY AND SUBSPECIALTY WITH RESEARCH ACTIVITIES over a six to seven-year period of training time." For other details, please see: https://students-residents.aamc.org/md-phd-dual-degree-training/training-physician-scientist-internal-medicine

In general, how important do YOU think the following factors were in obtaining a PSTP interview?

22. USMLE (Step 1/2CK) Scores

Mark only one oval.

	1	2	3	4	5	
Not Important						Very Important

23. Total number of peer-reviewed publications

Mark only one oval.

	1	2	3	4	5	
Not Important						Very Important

24. First-authored peer-reviewed Publication(s)

Mark only one oval.

	1	2	3	4	5	
Not Important						Very Important

25. Impact factor of peer-reviewed publication(s)

Mark only one oval.

	1	2	3	4	5	
Not Important						Very Important

26. History of prior extramural funding (F30, etc…)

Mark only one oval.

	1	2	3	4	5	
Not Important						Very Important

27. A personal connection with current faculty/program (self or advisor)

Mark only one oval.

	1	2	3	4	5	
Not Important						Very Important

28. Personal Statement

Mark only one oval.

	1	2	3	4	5	
Not Important						Very Important

29. Diversity

Mark only one oval.

	1	2	3	4	5	
Not Important	◯	◯	◯	◯	◯	Very Important

30. How much total time would you estimate spending on your application materials (in hours)?

____

31. To how many PSTPs did you apply?

____

32. To how many residency programs (PSTP or otherwise) did you apply, in total?

____

PSTP Interviews (3/6)

Physician-scientist training programs (PSTPs) refer to residency tracks that "integrate clinical training in internal medicine RESIDENCY AND SUBSPECIALTY WITH RESEARCH ACTIVITIES over a six to seven-year period of training time." For other details, please see: https://students-residents.aamc.org/md-phd-dual-degree-training/training-physician-scientist-internal-medicine

33. How many PSTP interview invitations did you receive?

____

34. How many PSTP interviews did you cancel?

____

35. If you canceled a PSTP interview, why?

Check all that apply.

- [ ] Overlapping Interview date(s)
- [ ] Already had enough interviews
- [ ] Lost interest in the program(s)
- [ ] Other: _______________________

36. What is your overall opinion of VIRTUAL interviewing?

Mark only one oval.

	1	2	3	4	5	
Strongly Dislike	◯	◯	◯	◯	◯	Strongly Like

37. Overall, how satisfied were you with program-organized interactions with residents (pre-interview social, interview day, second-look, etc...)?

Mark only one oval.

	1	2	3	4	5	
Not satisfied	◯	◯	◯	◯	◯	Completely satisfied

38. Anything else you would like to say about your PSTP interview experience?

______

Post-Interview Season (4/6)

Physician-scientist training programs (PSTPs) refer to residency tracks that "integrate clinical training in internal medicine RESIDENCY AND SUBSPECIALTY WITH RESEARCH ACTIVITIES over a six to seven-year period of training time." For other details, please see: https://students-residents.aamc.org/md-phd-dual-degree-training/training-physician-scientist-internal-medicine

39. Did any PSTPs initiate communication with you after interviews?

Check all that apply.

- [ ] Email
- [ ] Phone call
- [ ] Virtual meeting
- [ ] Nothing
- [ ] Other: ______

40. Do you think post-interview communication initiated by a program influenced your ranking decision?

Mark only one oval.

	1	2	3	4	5	
Definitely Not	◯	◯	◯	◯	◯	Definitely

41. At any point, did you feel pressured or incentivized to share your ranking preferences with a program?

Mark only one oval.

- ◯ Yes, explicitly
- ◯ Yes, implied
- ◯ No

42. Which, if any, of the following NRMP codes did any PSTPs violate? If needed, please refer to https://www.nrmp.org/policy/match-code-of-conduct-programs/

Check all that apply.

- [ ] Ensure an interview experience that is safe, respectful, and free of harmful bias.
- [ ] Maintain ethical behavior during recruitment (e.g. no recording interviews)
- [ ] Refrain from asking illegal questions (e.g. regarding race, marital/parental status, or sexual orientation)
- [ ] Fully Disclose Pertinent Information to Applicants (i.e. criteria used for vetting applications)
- [ ] Respect an applicant’s right to privacy and confidentiality (ranking preferences, other programs etc...)
- [ ] Do not require second visits
- [ ] Limit post-interview communication, and avoid misleading statements about ranking status
- [ ] Programs Rank with integrity (i.e. applicant's merit and alignment with the institution/program)

43. Did you visit with any PSTPs (or surrounding area) IN-PERSON following your interview?

Mark only one oval.

- Yes, and I met with someone affiliated with the PSTP
- Yes, but I did not meet with anyone affiliated with the PSTP
- No, but I wanted to visit
- No, and I preferred not to visit
- Other: ____________________

The Match (5/6)

For the purpose of this survey, physician-scientist training programs (PSTPs) refer to residency tracks that integrate clinical training in internal medicine RESIDENCY AND SUBSPECIALTY with research activities over a six to seven-year period of training time. For other details, please see: https://students-residents.aamc.org/md-phd-dual-degree-training/training-physician-scientist-internal-medicine

44. Where on your rank list did you match?

____

45. Did you match into a PSTP?

Mark only one oval.

- Yes
- No

46. In which state is your residency program located?

Mark only one oval.

- Alabama
- Alaska

- Arizona
- Arkansas
- California
- Colorado
- Connecticut
- Delaware
- Florida
- Georgia
- Hawaii
- Idaho
- Illinois
- Indiana
- Iowa
- Kansas
- Kentucky
- Louisiana
- Maine
- Maryland
- Massachusetts
- Michigan
- Minnesota
- Mississippi
- Missouri
- Montana
- Nebraska
- Nevada
- New Hampshire
- New Jersey
- New Mexico
- New York

- North Carolina
- North Dakota
- Ohio
- Oklahoma
- Oregon
- Pennsylvania
- Rhode Island
- South Carolina
- South Dakota
- Tennessee
- Texas
- Utah
- Vermont
- Virginia
- Washington
- West Virginia
- Wisconsin
- Wyoming

47. How important were research opportunities/community in your ranking preferences?

Mark only one oval.

	1	2	3	4	5	6	7	
Not Important								Most Important

48. How important was the institution's reputation in your ranking preferences?

Mark only one oval.

	1	2	3	4	5	6	7	
Not Important								Most Important

49. How important was the resident community when entering your ranking preferences?

Mark only one oval.

	1	2	3	4	5	6	7	
Not Important								Most Important

50. How important was the program support & leadership when entering your ranking preference

Mark only one oval.

	1	2	3	4	5	6	7	
Not Important								Most Important

51. When entering your ranking preferences, how important was it for a PSTP to guarantee fellowship?

Mark only one oval.

	1	2	3	4	5	6	7	
Not Important								Most Important

52. How important was institutional family/child/personal support (proximity to family, health insurance, daycare, lactation room, etc…) when entering your ranking preferences?

Mark only one oval.

	1	2	3	4	5	6	7	
Not Important	◯	◯	◯	◯	◯	◯	◯	Most Important

53. How important was “gut feeling” when entering your ranking preferences?

Mark only one oval.

	1	2	3	4	5	6	7	
Not Important	◯	◯	◯	◯	◯	◯	◯	Most Important

Possible Modifications (6/6)

54. What was the most stressful aspect of your residency application?

Mark only one oval.

- ◯ Choosing where to apply
- ◯ Completing the application
- ◯ Waiting for interviews
- ◯ Responding to interview requests
- ◯ Interviewing
- ◯ Post-interview communication
- ◯ Entering rank list
- ◯ Waiting for match results
- ◯ Other: ______________________

55. If you could change only one thing about PSTP applications & interviewing, what would it be?

____
____
____
____
____

56. How would you feel if programs coordinated their interview invitations and scheduling to avoi conflicting dates (item #1 on figure)?

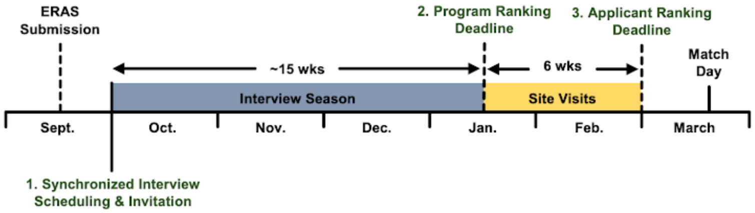

Mark only one oval.

	1	2	3	4	5	
Strongly Dislike	◯	◯	◯	◯	◯	Strongly Like

57. How would you feel if PSTPs were required to submit final rank lists a few weeks BEFORE applicants? (i.e. the "site visits" below, between #2 and #3)

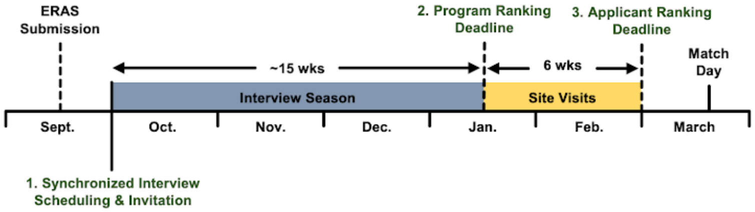

Mark only one oval.

	1	2	3	4	5	
Strongly Dislike	◯	◯	◯	◯	◯	Strongly Like

58. How would you feel if PSTPs offered purely non-evaluative site visits?

Mark only one oval.

	1	2	3	4	5	
Strongly Dislike	◯	◯	◯	◯	◯	Strongly Like

59. Should PSTPs participate in "The Match?"

Mark only one oval.

- ◯ Yes
- ◯ No

60. Any last thoughts/suggestions?

This content is neither created nor endorsed by Google.

Google Forms
